# Doxorubicin uptake in ascitic lymphoma model: resistance or curability is governed by tumor cell density and prolonged drug retention

**DOI:** 10.7150/jca.46066

**Published:** 2020-09-19

**Authors:** Gintaras Zaleskis, Sima Garberytė, Božena Pavliukevičienė, Gintaras Valinčius, Dainius Characiejus, Mykolas Mauricas, Jan Aleksander Kraśko, Karolina Žilionytė, Margarita Žvirblė, Vita Pašukonienė

**Affiliations:** 1 Laboratory of Immunology, National Cancer Institute, Vilnius, Lithuania.; 2 Institute of Biochemistry, Life Sciences Centre, Vilnius University, Vilnius, Lithuania.; 3 Department of Immunology, State Research Institute Centre for Innovative Medicine, Vilnius, Lithuania.

**Keywords:** chemotherapy resistance, anthracycline, doxorubicin, drug uptake, efflux, ascitic tumor, myelosuppression

## Abstract

**Background/Aims:** Chemotherapy resistance of malignancies is a universal phenomenon which unfavorably affects therapeutic results. Genetic adaptations as well as epigenetic factors can play an important role in the development of multidrug resistance. Cytotoxic drug content in plasma of cancer patients is known to variate up to one hundred-fold regardless of the same dose injected per m^2^ body surface. The relationship between plasma concentrations, tissue uptake, and chemotherapy response is not completely understood. The main objective of this study was to investigate how the identical dose of Doxorubicin (Dox) can result in a different therapeutic response pattern depending on tumor size.

**Study Design:** The study was performed on ascitic EL4 lymphoma in an exponential growth phase focusing on the rapidly changing tumor susceptibility to the Dox treatment. Well distinguishable tumor response patterns (curability, remission-relapse, resistance) were selected to unveil Dox intratumoral uptake and drug tissue persistence. Intratumoral Dox content within peritoneal cavity (PerC) in conjunction with systemic toxicity and plasma pharmacokinetics, were monitored at several time points following Dox injection in tumor bearing mice (TBM) with differing patterns of response.

**Results:** Following intraperitoneal (i.p.) transplantation of 5x10^4^ EL4 lymphoma cells rapid exponential proliferation with ascites volume and animal mass increase resulted in median survival of 14.5 days. The increase in tumor cell mass in PerC between day 3 and day 9 was 112.5-fold (0.2±0.03 mg vs 22.5±0.31 mg respectively). However, tumors at this time interval (day 3 to day 9 post-transplantation) were relatively small and constituted less than 0.05% of animal weight. An identical dose of Dox (15 mg/kg) injected intravenously (i.v.) on Day 3 lead to a cure whereas a TBM injected on day 9 exhibited resistance with a median survival time no different from the untreated TBM control. Injection of Dox resulted in noticeable differences of cellular uptake in PerC between all three groups of TBM (“cure”, relapse”, “resistance”). Larger tumors were consistently taking up less Dox 60 min after the 15 mg/kg i.v. bolus injection. Higher initial uptake resulted also in longer retention of drug in PerC cells. The area under the concentration curve in PerC cells AUC_0-10d_ was 8.2±0.57 µg/g x h, 4.6±0.27 µg/g x h and 1.6±0.02 µg/g x h in “cure”, “relapse” and “resistance” TBM respectively (*p*<0.05 “relapse” vs “cure” and *p*<0.001 “resistance” vs “cure”). No differences in plasma Dox pharmacokinetics or systemic hematological effects were observed in TBM following a single i.v. Dox push. Hematologic nadir was tested on day 2 and subsequent hematologic recovery was evaluated on day 10 following Dox administration. Hematologic recovery on day 10 coincided with complete drug efflux from PerC and rising tumor cell numbers in PerC of “relapse” TBM. Myelosuppression and hematological recovery patterns were identical in all surviving animal groups regardless of the tumor size on the day of Dox injection.

**Conclusions:** Within a few days of exponential tumor growth, an identical dose of Dox produced dramatically different responses in the TBM with increasing resistance. Systemic toxicity and plasma pharmacokinetics were indistinguishable between all TBM groups. Initial uptake in tumor cells was found to be consistently lower in larger tumors. Drug uptake in tumor cells was regulated locally - a phenomenon known as inoculum effect *in vitro*. The duration of drug retention in cells was directly related to initial cellular uptake. The magnitude of Dox cellular retention could potentially play a role in determining tumor remission and relapse.

## Introduction

Chemotherapy resistance and tumor relapse greatly affects the risk of death in cancer patients 1-3. Mainstream research on chemotherapy resistance in tumors call attention to genetically mediated mechanisms that lead to modified susceptibility to anticancer drugs. The concept of “drug resistant mutations” has been advocated since the 1950s 4 and it explains the curability of small tumors to some extent. However, some observations suggest that mutations might be just one factor, and possibly not a decisive one, in promoting the proliferation of resistant tumor cells 5-7. Cancer chemotherapy obviously spares some malignant cells even in the absence of resistance mutations. Therefore, several non-genomic factors that might trigger resistance were in focus during recent investigations, including: drug gradients in relation to blood vessels 8, regions of hypoxia 9, abnormalities of tumor vascularization 10, interstitial pressure 11, abundance of cellular debris 12 and some other phenomena. All these studies theoretically imply that higher injected doses must result in better therapeutic outcomes. Yet the relationship between dose injected, plasma concentrations, and tumor response rates is poorly understood 13. Higher plasma drug concentration does not inevitably mean higher tumor drug uptake or better therapeutic outcomes. Furthermore, plasma drug concentration in individual patients may vary 10-100-fold despite patients receiving the same dose of drug per m^2^ body surface 14-17. Higher plasma concentrations, only in some studies, resulted in better therapeutic results 14, 16, 18, 19. We explored the EL4 ascitic lymphoma model to demonstrate that Dox pharmacokinetics, systemic drug uptake, and hematologic toxicity were unrelated to therapeutic response. Tumors exhibited different patterns of therapeutic response based solely on local intratumoral Dox uptake and retention at different stages of growth. Initial uptake and forthcoming retention expressed as AUC of cellular Dox in the tumor was the only factor governing the curability or resistance in our model.

## Materials and Methods

### Animals

Female C57BL/6LNCr mice, aged 12 to 14 weeks, were used and cared for in accordance with the Guide for the Care and Use of Laboratory Animals. All research protocols were approved by the Institutional Animal Care Committee. Animals had *ad libitum* access to pelleted feed, food supplements, and water. All animals were determined to be specific pathogen free.

### Drugs and reagents

Doxorubicin hydrochloride was purchased from Ebewe (Unterach, Austria), daunorubicin hydrochloride, analytical grade chloroform, HPLC grade acetonitrile, orthophosphoric acid and hydrochloric acid were purchased from Sigma Chemical Company (St. Louis, Mo, USA). HPLC grade methanol was purchased from Carl ROTH (Karlsruhe, Germany).

### Tumor model, treatment and experimental groups

Mice were given inoculations intraperitoneally under aseptic conditions of 0.2 ml of pooled fresh ascitic fluid (5×10^4^ EL4 lymphoma cells per mouse). We were not able to achieve curability or immune rejection via the tapping procedure as described in studies with EL4 line 20. Ascitic EL4 was highly aggressive and untreated animals survived no longer than 16 days after transplantation of the tumor. The cell counting was done within fresh ascitic fluid in a hemocytometer. The total number of ascitic cells was obtained by multiplying the volume of ascites by the number of cells/cu mm. Differential counts were performed on smears stained by May-Grünwald technic, with 200 cells being counted on each slide. The absolute number of each cell type in the ascitic fluid was obtained by multiplying the percentage yielded by the differential count with the total number of cells in the ascitic fluid. Dox was injected i.v. via tail vein as a single push with a dose of 15 mg/kg. Groups were labeled based on the tumor size and the day treatment was given, namely: 1) “Cure” tumor bearing mice (TBM) - for the animals that received a Dox injection on day 3 after tumor transplantation, where a significant proportion of these animals (60%) survived more than 60 days and were designated as “cure“; 2) “Relapse” TBM - for the animals that received identical Dox injections on day 5, where the animals showed temporal reduction in tumor size but eventually succumbed to the tumor; 3) “Resistance” TBM, for animals that received an identical dose of Dox on day 9, where these mice did not show any advantage of survival compared to untreated control mice. Where appropriate, control mice were included and were either left untreated or received saline vehicle solution. The number of surviving mice in each experimental group was checked twice daily until day 30, then checked daily until day 60. All mice that died had an evident ascitic tumor except for those in “cure” TBM group.

### Determination of total volume of ascites

The body weight of each mouse was measured using an electronic balance every day (precision: 0.1 g). The mice were killed by cervical dislocation, the peritoneal cavity was opened, and all fluid was withdrawn with a syringe with special care taken to leave coagulated material in place. The peritoneal cavity was then gently and thoroughly dried with a cotton sponge after which each mouse was again weighed. By subtraction of the "dry" body weight (after removal of the fluid by syringe and cotton sponge) from the "wet" weight (animal's weight prior to death), the amount of ascitic fluid expressed in grams was obtained. For control mice and those with a minimal amount of ascites, the weight change of the cotton sponge after soaking it with peritoneal fluid was recorded.

### Blood sampling, plasma preparation, complete blood counts (CBC)

Blood samples (125 µL) were taken from the retroorbital plexus (blank sample) after the drug administration at several time points (5 min, 15 min, 30 min, 60 min, 6 h 12 h, 48 h and 72 h) in tubes containing EDTA as an anticoagulant. Each blood sample was gently inverted several times to ensure complete mixing with the anticoagulant. After centrifugation at 5000 × g for 10 min, plasma samples were separated and stored at -20 °C until analysis. CBC were analyzed using ABX Micros ESV60 (Horiba).

### Spleen and thymus suspension preparation and flow cytometry analysis

Spleen and thymus were excised from Dox treated and untreated mice then forced through coarse and fine stainless-steel screens to make single cell suspensions. For flow cytometry (FCM) analysis cells were adjusted to 10^6^ cells/ml concentration and kept on ice until evaluation with FACS LSRII (Beckton Dickinson) flow cytometer. Differences in mean channel fluorescence (dMCF) at 575/26 nm after 488nm excitation were recorded in log scale. EL4 lymphoma cells were exhibiting distinct light scatter characteristics as compared to host peritoneal exudate cells. The differences between spleen and thymus organ weight were expressed as a ratio to body weight. Following preservation in 10% neutral buffered formalin, tissues were embedded in paraffin then sectioned (4 µm thickness). Sections were stained with hematoxylin and eosin then examined by light microscopy.

### Pharmacokinetics

Microvolume method 21 was applied in this study for plasma Dox analysis. 50 µL of internal standard solution (800 ng/mL Daunorubicin hydrochloride) was added to 60 µL of a plasma sample and vortex-mixed for 30 seconds. The extraction of the drug was performed by adding 900 µL of a chloroform/methanol mixture (4:1, v/v). After vortex mixing for 10 min and centrifugation (10 min, 10000 × g), the organic phase was collected, transferred to a clean tube then evaporated to dryness under a stream of nitrogen. Dry residue from the plasma was dissolved in 60 µL of mobile phase, and after centrifugation for 5 min (10000 × g), injected into the chromatographic column. For cellular suspensions, the HPLC method described earlier was explored 22. Briefly, 10^6^ cells in water were sonicated for 30 seconds (Branson 450 Sonifier, duty cycle 90%). After the addition of 111 µl of 1N HCl, samples were vortexed for 1 min, allowed to sit on ice for 15 min, then vortexed again and centrifuged at 600 × g for 5 min. Aliquots of supernatants (150 µl) were kept frozen until assayed. The area under the concentration curve AUC_0-10d_ for Dox content in peritoneal cavity (PerC) cells were calculated by trapezoid rule from the values obtained during 10 day (240 hours) period after Dox administration.

### Chromatographic conditions

High performance liquid chromatography (HPLC) analysis was performed on a Perkin Elmer system that consisted of a Flexar binary LC pump, a Kit - Flexar 3 CHNL VAC degasser, a Flexar LC autosampler, and Flexar fluorescence detector (Xenon lamp). The chromatography data was acquired by Chromera software from Perkin Elmer. The chromatography separation was performed on a Brownlee Bio C18 column (4.6 × 150 mm, 5 μm particle size, Perkin Elmer, Shelton, USA). The optimum mobile phase consisting of acetonitrile and water (32:68, v/v), was pH adjusted to 2.6 with 85% orthophosphoric acid. Samples were delivered via isocratic flow at a rate of 0.25 mL/min. The column temperature was maintained at 37°C and excitation and emission wavelengths were set at 475 and 555 nm respectively. The injection volume was 50 µL.

### Statistical analysis

All the results are presented as means and standard error (mean ± SE). The differences in the mean values among different groups were determined by a one-way analysis of variance with Tukey post hock test using Statistica 12.0 (TIBCO Software Inc.). Significance was considered at values of *p*<0.05.

## Results

**Figure 1:**
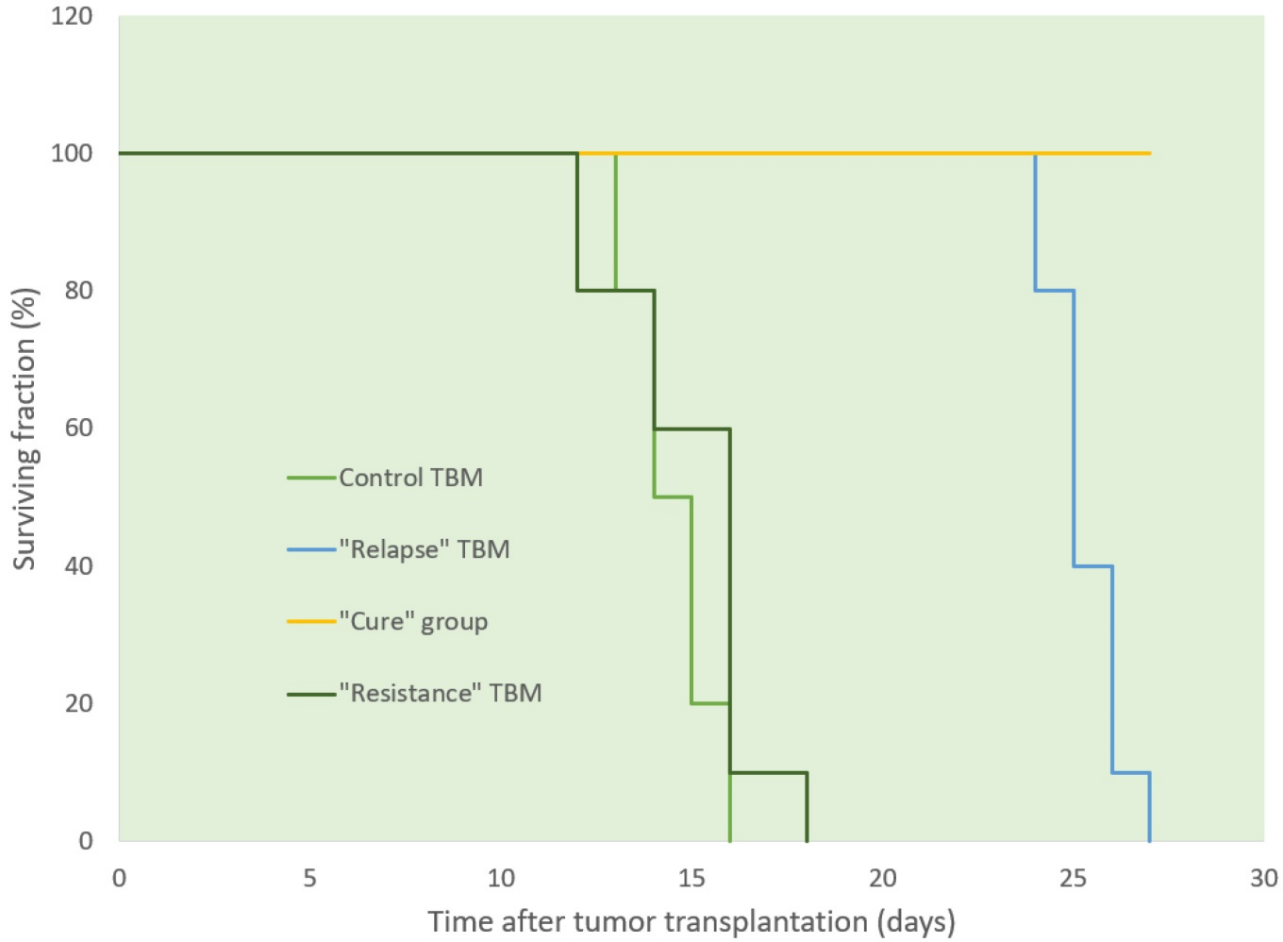
Survival of TBM following i.v. administration of Dox at different stages of tumor growth. Sixty per cent of mice in “cure” group (yellow line) survived more than 60 days after tumor transplantation (data not shown).

**Figure 2:**
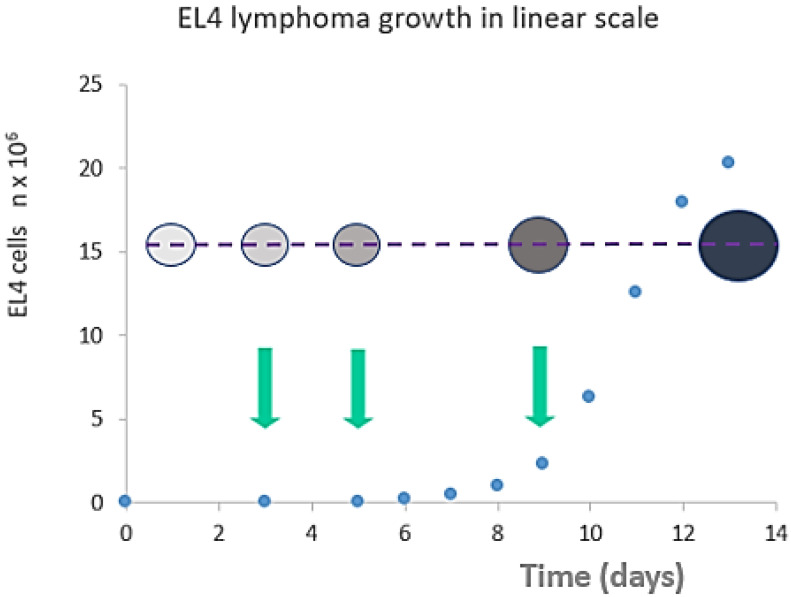
Tumor size (cell counts - blue dots) vs animal weight (bubble diameter) vs cell density (bubble color intensity) in peritoneal exudate cells following tumor transplantation. Arrows indicate selected treatment days (day 3 “cure”, day 5 “relapse” or day 9 “resistance”) for Dox injection. Unlike the late stage (day 13) tumors, early stage (days 1-9) tumors did not exhibit a significant increase in mice weight or an increase in ascites volume. Despite this, the response to Dox administration is significantly different at different points in time as well as tumor cell density in the early stage.

**Figure 4:**
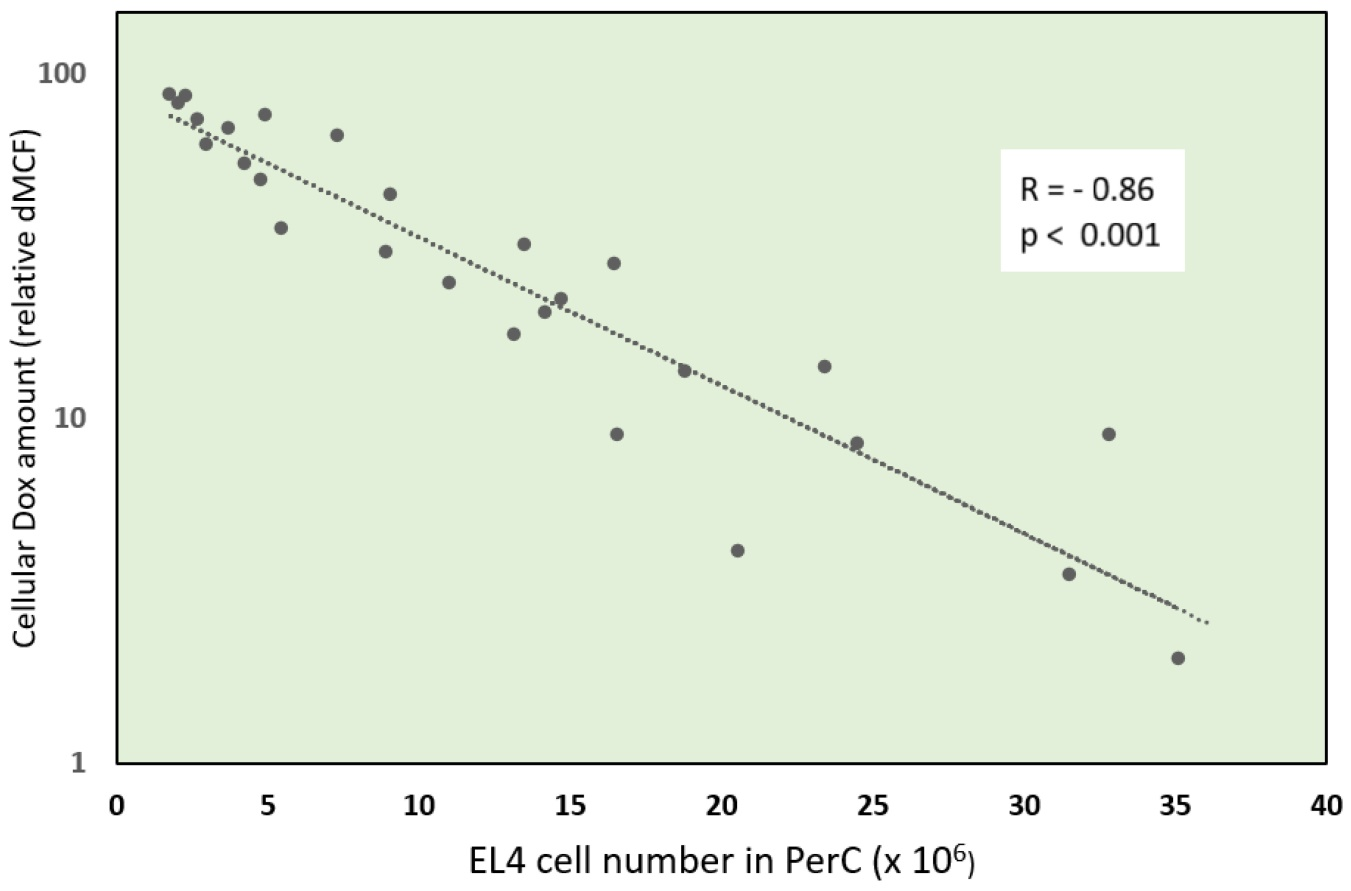
Negative correlation (*r*= -0.86; *p*<0.001) of cellular Dox amount and tumor size - EL4 cell number in peritoneal cavity (PerC). PerC cells of individual mice were examined by flow cytometry for Dox uptake - relative difference in mean channel fluorescence (dMCF) and plotted versus EL4 cell number.

Ascitic EL4 lymphoma exhibited a typical rapid proliferation pattern in PerC. Median survival time of untreated TBM was 14.5 days (**Fig. 1**). Significant animal weight increase (1.72-fold) was observed during tumor progression. Mouse weight ranged from 20.0±0.87 g before EL4 transplantation to 34.4±1.52 g on day 13 of tumor growth. Lymphoma cell numbers increased exponentially (**Fig. 2**) from 0.05×10^6^ on day 0 to 203×10^6^ on day 13. Ascitic volume increase, relative host cell number decrease, and tumor cell density surge were accompanying the progression of EL4 in PerC. While tumor cell mass increased 4,060-fold, total ascites volume increased 62-fold: from 0.2 ml to 12.4 ml during 13 days of tumor growth. Tumor cells in suspension within the PerC contributed to only 0.2 g of intraperitoneal cell mass at late stage tumor growth (day 13). Presence of solid tumor deposits laying on serous cavity surfaces and aggregates also contributed to total tumor and animal mass at day 13. However, solid subcutaneously implanted EL4 tumors of this size and at this stage (day 13) were not lethal (data not shown). Rapid fluid accumulation beginning on day 10 was a characteristic feature of ascitic tumor growth. Substantial influx of RBC was noticeable starting from day 10. Absence of platelets accompanying RBCs influx implies the presence of activated clotting and aggregation on serous surfaces. These coagulation and permeability characteristics were reported in other ascitic models 30. The time between day 1 and 9 revealed relatively steady intraperitoneal conditions (no hemorrhage or extensive aggregation), yet disclosing different patterns of chemotherapy response. TBM administered 15 mg/kg of Dox on day 3 (EL4 cell number 0.15×10^6^, cell density 0.6×10^6^/ml) survived >60 days. Six out of 10 animals reached this time point, while the remaining 4 did not show ascitic tumor presence. This group of TBM was designated as the “cure” group. Mice administered with the same dose of Dox on day 5 showed signs of remission which resulted in a median survival time of 26 days. No TBM in this group survived. This group was designated as the “relapse” group. TBM treated on day 9 showed no signs of response, with their survival rates no different from the untreated control mice (14 days). TBM injected on day 9 (EL4 cell number 22.7×10^6^, density 26.8×10^6^/ml) were designated as “resistant”. Dox uptake in PerC cells was found to have negative correlation with tumor size (*r* = - 0.86; *p*<0.001; **Fig. 4**). Although the difference in tumor size between day 3 (“cure” group) and day 9 (“resistance” group) was found to be an increase by 112.5-fold, the actual tumor weight compared to mouse weight remained miniscule - 0.045% (**Table 1**). Sixty minutes after Dox administration, cellular drug uptake and forthcoming retention for 10 days (calculated as AUC_0-10 d_) was observed in all TBM. Dox uptake by individual cells and AUC_0-10d_ were found to have an inverse relationship to tumor size in PerC (**Table 1**).

Plasma drug pharmacokinetics revealed rapid clearance with the peak plasma concentration at 5 min, clearance Cl = 3.1 l × kg^-1^ h^-1^ and half-time t_½_ = 28.2 h. Plasma drug presence was below detection limit at 72 h point. No significant differences were observed in the plasma of control mice and TBM. However, in long term surviving mice, the drug was retained in cellular samples of PerC, spleen, and thymus up to 18 days following i.v. administration of 15 mg/kg Dox. Cellular Dox retention during this period was detectable through both HPLC and FCM. No drug was detectable at 60 day time point in long term survivors although we could not exclude the presence of some residual Dox in cellular samples detectable by potentially more sensitive method.

Dox-induced systemic myelosuppressive effects did not differ among the groups of TBM regardless of tumor size variability (**Table 2**). The Dox-induced nadir was tested on day 2 after i.v. administration. We observed in all Dox treated mice that the total white blood cell (WBC), absolute lymphocyte, and monocyte counts were significantly lower when compared to untreated control mice. A significant decrease in thymus and spleen weight was also observed. A histological evaluation of the thymus showed that Dox treatment led to a depletion of thymus cortical cells and a relative increase in the number of Hassall's corpuscles. On day 10, post Dox injection, suppressed hematopoietic cells recovered both in “cure” and “relapse” groups as well as in control mice. No recovery in organ weight was seen in the thymus at 10 days, post Dox injection.

There were no measured differences in mean cell volume (MCV) or mean platelet volume (MPV) values in Dox treated mice at any point in time. These parameters were recently reported to play a significant role in some cases of chemotherapy response 23-26. We therefore tested these clinical parameters to potentially reveal their role in systemic hematopoietic effects. The recovery of hematological myelosuppression coincided with nearly complete clearance of cellular Dox detectable in the spleen and thymus of surviving TBM on day 10 after Dox injection. The “cure” TBM displayed a significantly higher AUC_0-10d_ of cellular Dox as compared to “relapse” or “resistance” TBM. In addition to Dox efflux and hematologic recovery, the “relapse” TBM showed detectable tumor cell counts (29.5×10^6^) at the recovery time point (day 10 after Dox injections, day 15 after tumor transplantation).

We also evaluated the anti-tumor and myelosuppression activity of temperature degraded Dox. Dox exposed to 37 °C for a maximum of 120 days revealed a marginal, but still detectable, profile of myelosuppression activity as compared to fresh Dox (**Table 1**). HPLC chromatograms of degraded Dox samples showed a 16.8 times lower peak height, appearing at 11.1 minutes, compared to fresh Dox. There was no significant anti-tumor activity when degraded Dox was injected during early stages of lymphoma tumor (data not shown). The dose injected (15 mg/kg degraded Dox) corresponds to less than 1 mg/kg of fresh Dox. Our previous observations revealed that fresh Dox (1 mg/kg i.v.) did not cure lymphoma at any stage, including day 1. Degraded Dox presented a darker coloration of the solution (2 mg/ml) when stored at 37 °C in tightly closed vials for 120 days. We assume that long term tissue persistence of indwelling Dox might not entirely abolish its cytotoxic capabilities in the tumor microenvironment.

## Discussion

Efforts are often made to identify a genetic marker responsible for chemotherapy resistance, on the assumption that the correlation between tumor size and curability is related to the mutation rate. We explored the EL4 model to demonstrate that curability and resistance might also be dependent on a different mechanism, namely Dox uptake level and persistence in tissues and individual cells. Human tissues and cells are known to internalize up to 100 times more Dox compared to the maximum plasma concentration following i.v. administration 27. Moreover, Dox and other cytotoxic drugs are sometimes retained in the tissues of patients for several years after the accomplishment of treatment 28, 29. We attempted to determine if the cellular retention of Dox could be a significant factor in slowing down the recurrence of a tumor or curing TBM permanently. Dox content in the tumor's tissue shortly after injection was directly related to the EL4 lymphoma treatment response. We investigated only the early avascular phase (days 3 to 9 after transplantation) since late stage tumors manifest hemorrhagic high-volume ascites with systemic toxicities. During this initial phase, tumor aggregates or deposits on the peritoneal wall were negligible and potentially did not influence drug uptake in ascitic cells. Modest changes in cell numbers and cell density during the early avascular phase dramatically affected uptake, retention, and treatment response to EL4 ascitic lymphoma.

Plasma pharmacokinetics of Dox and systemic myelosuppressive effects did not differ significantly between different tumor sizes in the TBM. An increase in mice weight due to ascites accumulation was observed during the terminal stage of TBM (day 10 and thereafter). Terminal stage TBM had only 0.2 g of tumor suspended in ascitic fluid; therefore, the weight increase (by ~13 g on average) can be mostly attributed to an accumulation of ascites and solid deposits in the fluid compartment. Unlike the ascites, a solid subcutaneous EL4 tumor of the same size (0.2 g) was not lethal to the mice. Conversely, the ascites tumor was rapidly leading to death of the mice. Accumulation of lethal ascites is a complex phenomenon which is significantly influenced by vascular permeability and other factors in TBM 30. The density of tumor cells in the PerC increased by almost three orders of magnitude faster compared to ascites volume. The increasing density of suspended cells in the PerC was directly related to increased resistance. It is usually assumed that ascitic fluid accumulation results mostly from hyperpermeability of the blood vessels that line serous cavities 30, 31. Increasing hyperpermeability of plasma proteins demonstrated in some studies 30 would also imply that Dox permeation through the peritoneal line vasculature must be increased as the tumor progressed. In our EL4 model however, the larger tumors consistently showed less drug accumulation per cell compared to the smaller ones. Larger tumors were also exhibiting higher cellular density.

In fact, reduced Dox uptake per cell in suspension with increasing cellular density might be explained by the inoculum effect, which was described for leukemia cells exposed to anthracyclines *in vitro* 32. We hypothesize that the inoculum effect might play an important role in the response to Dox in the EL4 lymphoma model. There is indirect evidence that the inoculum effect might be not limited to *in vitro* occurrences only. For instance, high chemotherapy resistance of acute leukemias with high WBC counts might also be explained by the inoculum effect. It is generally considered that most patients with a high WBC count have genetic mutations leading to a more aggressive leukemia 33. However, the non-genetic factor, as simple as leukemia cell abundance (which leads to a highly heterogenous distribution of drug between circulating cells) might initially lead to resistance and plasma drug depletion in peripheral blood. Of particular note is the fact that patients with acute leukemia have a large interindividual variation in WBC counts accompanied by highly variable plasma concentrations of anthracyclines after drug infusion 18,19.

The widely known inoculum effect implies that *in vitro* cellular drug uptake is inversely correlated to cell numbers. The uptake might be so intense that a drug is sometimes depleted from the media 32. The PerC could be considered a closed system for a short duration of time following i.v. injection. The 5 sec exposure of cells to Dox *in vitro* was demonstrated to be sufficient to internalize a measurable amount of Dox 34. Dox uptake in peritoneal cells in our model was noticeable as early as 5 min after injection of the drug. Afterwards, cells retaining Dox showed a slow stable decline of cellular drug content with the duration of cellular retention amounting to several weeks. Dox cellular AUC_0-10d_ of TBM exhibiting relapse was significantly lower compared to that seen in TBM which were cured from the tumor. We hypothesize that Dox persistence in cells and tissues was playing an important role in preventing ascitic lymphoma from relapse. Tissue-bound or DNA-intercalated Dox might be degrading and losing cytotoxic potential during long term tissue in-dwelling. It is generally assumed that reconstituted commercial Dox (2 mg/ml) is stable for a maximum of 7 days at room temperature and 15 days under refrigeration (2 °C to 8 °C). We therefore tested Dox decay and residual cytotoxicity after a maximum 120-day exposure to 37 °C. During this time period, the Dox peak which is characteristic of a fresh sample could still be detected in HPLC histograms and the reduction of potentially intact drug fraction was 16.8-fold. Injecting 15 mg/kg of this Dox decay product did not affect tumor growth in our model. However, it still contained a small part of its original cytotoxic and myelosuppressive capacity as could be demonstrated by CBC counts. This means that tissue-bound and partially decayed Dox can still elicit its cytotoxic effect in the vicinity of tumor cells. The long-term tissue retention of cytotoxic drugs, including Dox, was studied by others in relation to neuro and cardiac toxicities in cancer survivors 28, 29, 35. Our observations suggest that tissue retention of the drug for a prolonged period can play an important role in keeping tumor cells “dormant” or even eliminating them completely.

From the standpoint of tumor recurrence, we observed gradual extrusion of Dox from tumor and host cells for a period of 10-18 days after i.v. treatment. An active efflux mechanism is the main cause for multidrug resistance (MDR) of tumor cells. For instance, MDR1 expression is an important element of trans-membrane efflux pumps that push different substances out of cells 36. A variety of chemotherapeutic agents, including Dox, are known to be the substrates of p-glycoprotein 37,38. Overexpression of MDR1 was demonstrated to be crucial to both initial and induced chemotherapy resistance of tumor cells, resulting in increased resistance of cancer cells to cytotoxic drugs 39,40. Here we show that Dox was slowly eliminated from the tumor and host cells at a rather steady rate for a prolonged period *in vivo*. The tumor size and cell density in PerC were the only factors governing uptake and duration of retention as it was seen by cellular AUC_0-10d_ in our study. Classic tests for the demonstration of p-glycoprotein dependent efflux explore a short-term tracking technique *in vitro* 41,42. Cellular drug retention studies for a duration of several weeks are not feasible in these *in vitro* settings. Here we show that cellular internalization of Dox could last for several weeks in TBM. The backward reverse gradient is known to function because of a lower Dox concentration in the ascitic fluid and plasma in the efflux stage. Recurrence of EL4 lymphoma was consistently linked to substantial extrusion of Dox from peritoneal cells. The indirect evidence showing that Dox cellular persistence might be a decisive factor in eliciting cytotoxicity was demonstrated by others with host hematopoietic precursor cells 43. Short-lasting peak cellular Dox concentrations which occur after a bolus injection were not essential for the cytotoxic effect, in contrast to the slow and high level 'tightly-bound' cellular Dox retention. The “tight bound” Dox was not very prone to outward efflux, although 90% of cellular Dox could be attributed to a “loose bound” category after short exposure observations in this study. Although initial uptake and persistence were highly correlated in our EL4 leukemia model, we admit that precise Dox internalization and efflux mechanisms might be quite complex and cannot always be explained by initial uptake and reverse gradients only.

In this way a relatively modest increase in tumor size with no systemic physiological harm resulted in full scale chemotherapy response ranging from “resistance” to “cure”. This is not unexpected since curing effects were reported in murine lymphoma models depending on the tumor size and agent dose 4, 44. However, the modern concept implies that resistance must inevitably be related to the presence of genetic mutations within a population of tumors. This concept was postulated decades ago and remains basically unchanged. The inadequacy of these assumptions was already seen in the first theoretical calculations 44. In fact, these calculations concluded that “less than one cell” amongst one million tumor cells “must be resistant” to a cytotoxic drug.

The parallels of cytotoxicity and myelosuppression indicate that the availability of Dox in a tumor microenvironment might be monitored by WBC and neutrophil counts 45,46. However, its wide clinical implementation needs some further improvements. In our study, recovery of hematological parameters was noticeable about the same point when lymphoma relapsed. “Resistance” TBM showed WBC nadir at 48 h following Dox injection, but no significant antitumor effect was observed. It is clear that local intratumoral and systemic Dox effects are not always related. Clinical observations that hematologic toxicity is the best surrogate parameter for an adequate and curative dose 45,46 could not be confirmed in our findings. In our model tissue efflux of a temporarily retained Dox was consistently resulting in subsequent tumor relapse.

## Figures and Tables

**Figure 3:**
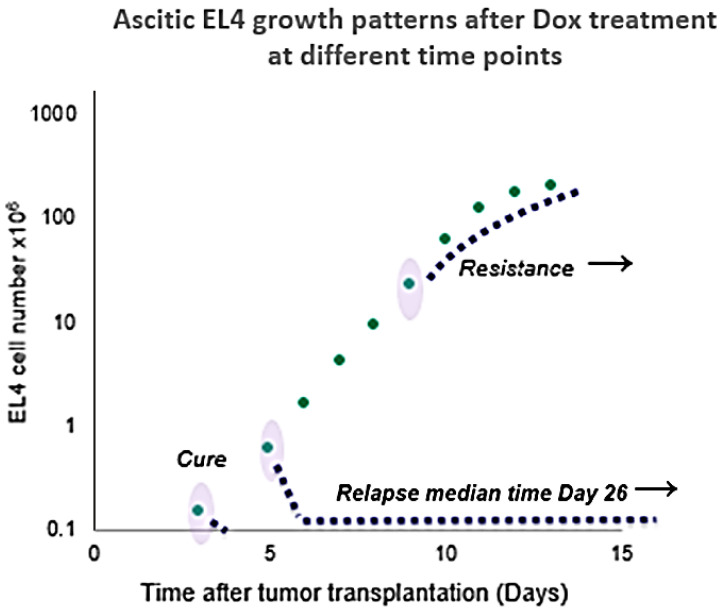
Response to Dox treatment at different points in time (days 3, 5, and 9) after tumor transplantation. Tumor cell counts are shown in log scale to visualize the miniscule difference in tumor size at the early stage of growth. Absolute cell numbers with standard error values are shown in the adjacent table. Table is adjacent to a scatterplot to indicate standard error of cell counts of all tested days. Dox injection days and corresponding tumor cell numbers are highlighted in the table.

**Table 1:** Dox concentration in plasma and peritoneal cavity cells 60 min after i.v. bolus administration of 15 mg/kg

Parameter	Animal groups			
	Control	'Cure' TBM	'Relapse' TBM	'Resistance' TBM
Plasma Dox (ng/µl)	0.25±0.050	0.25±0.066	0.22±0.054	0.21±0.049
Cellular PerC Dox (ng/10^6^ cells)	11±0.6	11±0.5	9±0.4*	7±0.8*
Cellularity PerC (n × 10^6^)	2.2±0.30	2.3±0.30	2.8±0.21	24.2±0.40**
EL4 cell number in PerC (n × 10^6^) or tumor mass (mg) (#)	N.A.	0.2±0.03	0.6±0.08*	22.5±0.31**
EL4 cellular density in PerC (10^6^/ml)	N.A.	0.6±0.14	1.8±0.96*	26.8±3.6**
Cellular Dox (dMCF) in EL4 cells (##)	N.A.	85±2.2	58±1.7*	11±2.5**
Animal body weight (g)	20.0±0.87	19.9±0.43	19.9±0.95	20.3±0.70
Ratio (%) of calculated tumor mass to animal weight	N.A.	0.001	0.003	0.045
Cellular Dox AUC_(0-10d)_ (µg/g × h)	8.3±0.49	8.2±0.57	4.6±0.27*	1.6±0.02**

*P < 0.05; **P < 0.01; N.A. - not applicable Dox - doxorubicin; PerC - peritoneal cavity; TBM - EL4 tumor bearing mice; dMCF - difference in mean channel fluorescence; AUC - Area under the concentration curve; #: Tumor mass calculations were made from the EL4 percentage values and PerC cellularity. One mg of tumor without ascitic fluid component equals 1 × 10^6^ cells; ##: EL4 gated cellular fluorescence increase (mean channel volume) 60 min after Dox i.v. bolus administration.

**Table 2:** Systemic hematopoietic effects (nadir and recovery) of Dox administered to control animals and tumor bearing mice. Complete blood cell counts (CBCs)

Blood sampling time (nadir, recovery or long-term outcomes)	CBC parameters							
	HGB (g/l)	MCV (fl)	PLT (K/μL)	MPV (fl)	WBC (K/μL)	LY (K/μL)	MO (K/μL)	GR (K/μL)
Control (no Dox)	142±8.4	46±0.6	751±70.7	6.8±0.61	7.5±0.67	6.0±0.70	1.1±0.15	0.3±0.06
**Day 2 post-injection (nadir)**								
Intact, Dox injected (#)	139±7.8	46±0.6	713±100.6	7.5±0.10	2.5±0.40**	1.3±0.25**	0.8±0.05*	0.4±0.12
“Cure” TBM	140±9.6	46±1.0	658±75.9	7.4±0.32	2.5±0.46**	1.2±0.35**	0.8±0.06*	0.5±0.10
“Relapse” TBM	143±6.0	46±0.6	639±121.0	7.3±0.26	2.6±0.25**	1.6±0.21**	0.7±0.06*	0.4±0.10
“Resistance” TBM	139±5.8	47±0.6	671±89.1	7.3±0.31	2.5±0.40**	1.7±0.70*	0.5±0.23*	0.3±0.15
**Day 10 post-injection (recovery)**								
“Cure” TBM	144±15.1	47±1.2	763±160.2	6.7±0.21	7.3±0.38	5.9±0.15	1.1±0.15	0.3±0.15
“Relapse” TBM	140±16.8	46±0.6	768±105.0	6.6±0.51	6.8±0.93	5.3±0.66	1.2±0.21	0.3±0.12
Day 60 long term survivors of “cure” TBM	146±6.7	47±0.1	830±157.4	6.7±0.40	7.7±1.12	5.8±1.16	1.2±0.10	0.4±0.12
Day 2 nadir of degraded Dox (##)	137±5.1	46±0.6	720±77.3	6.7±0.23	4.7±1.90*	3.3±1.25*	0.9±0.42	0.5±0.30

CBC, complete blood count; HGB, hemoglobin; MCV, mean cell volume; PLT, platelets, MPV, mean platelet volume; WBC, white blood cells; LY, lymphocytes; MO, monocytes; GR, granulocytes, TMB, tumor bearing mice. *p < 0.05; **p <0.01; (#) Day 2 nadir evaluated in intact mice injected i.v. with fresh Dox 15 mg/kg; (##) Day 2 hematological nadir has been evaluated in intact mice injected with degraded Dox. “Degraded” Dox was considered a specimen of drug kept at 37 °C for 120 days in darkness. Dox was injected to intact control mice.

## References

[1] Pokhriyal R, Hariprasad R, Kumar L (2019). et al. Chemotherapy Resistance in Advanced Ovarian Cancer Patients. Biomark Cancer.

[2] Epperla N, Badar T, Szabo A (2019). Postrelapse survival in diffuse large B-cell lymphoma after therapy failure following autologous transplantation. Blood Adv.

[3] Lafourcade A, His M, Baglietto L (2018). et al. Factors associated with breast cancer recurrences or mortality and dynamic prediction of death using history of cancer recurrences: the French E3N cohort. BMC Cancer.

[4] Skipper HE, Schabel Jr FM, Bell M (1957). et al. On the curability of experimental neoplasms. I. A-Methopterin and mouse leukemias. Cancer Res.

[5] Pisco AO, Huang S (2015). Non-genetic cancer cell plasticity and therapy-induced stemness in tumour relapse: 'What does not kill me strengthens me'. Br J Cancer.

[6] Brock A, Chang H, Huang S (2009). Non-genetic heterogeneity - a mutation-independent driving force for the somatic evolution of tumours. Nat Rev Genet.

[7] Patel KJ, Trédan O, Tannock IF (2013). Distribution of the anticancer drugs doxorubicin, mitoxantrone and topotecan in tumors and normal tissues. Cancer Chemother Pharmacol.

[8] Lankelma J, Dekker H, Luque FR (1999). Doxorubicin gradients in human breast cancer. Clin Cancer Res.

[9] Saggar JK, Tannock IF (2014). Activity of the hypoxia-activated pro-drug TH-302 in hypoxic and perivascular regions of solid tumors and its potential to enhance therapeutic effects of chemotherapy. Int J Cancer.

[10] Siemann D.W (2011). The unique characteristics of tumor vasculature and preclinical evidence for its selective disruption by tumor-vascular disrupting agents. Cancer Treat Rev.

[11] Böckelmann LC, Schumacher U (2019). Targeting tumor interstitial fluid pressure: will it yield novel successful therapies for solid tumors? J. Expert Opin Ther Targets.

[12] Chang J, Bhasin SS, Bielenberg DR (2019). Chemotherapy-generated cell debris stimulates colon carcinoma tumor growth via osteopontin. FASEB J.

[13] Bardin C, Veal G, Paci A (2014). Therapeutic drug monitoring in cancer-are we missing a trick?. Eur J Cancer.

[14] Frost BM, Eksborg S, Björk O (2002). Pharmacokinetics of doxorubicin in children with acute lymphoblastic leukemia: multi-institutional collaborative study. Med Pediatr Oncol.

[15] Palle J, Frost BM, Peterson C (2006). Nordic Society for Pediatric Hematology and Oncology. Doxorubicin pharmacokinetics is correlated to the effect of induction therapy in children with acute myeloid leukemia. Anticancer Drugs.

[16] Preisler HD, Gessner T, Azarnia N (1984). Relationship between plasma adriamycin levels and the outcome of remission induction therapy for acute nonlymphocytic leukemia. Cancer Chemother Pharmacol.

[17] Bogason A, Quartino AL, Lafolie P (2011). Inverse relationship between leukaemic cell burden and plasma concentrations of daunorubicin in patients with acute myeloid leukaemia. Br J Clin Pharmacol.

[18] Robert J, Rigal-Huguet F, Harousseau JL (1987). Pharmacokinetics of idarubicin after daily intravenous administration in leukemic patients. Leuk Res.

[19] Paul C, Liliemark J, Tidefelt U (1989). Pharmacokinetics of daunorubicin and doxorubicin in plasma and leukemic cells from patients with acute nonlymphoblastic leukemia. Ther Drug Monit.

[20] Apffel CA, Arnason BG, Twinam CW (1966). Recovery with immunity after serial tapping of transplantable mouse ascites tumours. Br J Cancer.

[21] Daeihamed M, Haeri A, Dadashzadeha S (2015). Simple and sensitive HPLC method for fluorescence quantitation of Doxorubicin in micro-volume plasma: applications to pharmacokinetic studies in rats. Iran J Pharm Res.

[22] Zaleskis G, Ho RL, Diegelman P (1994). Intracellular doxorubicin kinetics in lymphoma cells and lymphocytes infiltrating the tumor area *in vivo*: a flow cytometric study. Oncol Res.

[23] Jung HA, Kim H-J, Maeng CH (2015). et al. Changes in the mean corpuscular volume after Capecitabine treatment are associated with clinical response and survival in patients with advanced gastric cancer. Cancer Res Treat.

[24] Chang J, Lin G, Ye M (2019). Decreased mean platelet volume predicts poor prognosis in metastatic colorectal cancer patients treated with first-line chemotherapy: results from mCRC biomarker study. BMC Cancer.

[25] Jank BJ, Haas M, Dunkler D (2019). et al. Analysis of perioperative platelet indices and their prognostic value in head and neck cancer patients treated with surgery and postoperative radiotherapy: a retrospective cohort study. J. Clin. Med.

[26] Omar M, Tanriverdi O, Cokmert S (2018). Role of increased mean platelet volume (MPV) and decreased MPV/platelet count ratio as poor prognostic factors in lung cancer. Clin Respir J.

[27] Speth PAJ, Linssen PCM, Boezeman JBM (1987). et al. Cellular and plasma adriamycin concentrations in long-term infusion therapy of leukemia patients. Cancer Chemother. Pharmacol.

[28] Stewart DJ, Grewaal D, Green R (1993). et al. Concentrations of doxorubicin and its metabolites in human autopsy heart and other tissues. Anticancer Res.

[29] Gerl A, Schierl R (2000). Urinary excretion of platinum in chemotherapy-treated long-term survivors of testicular cancer. Acta Oncol.

[30] Nagy JA, Masse EM, Herzberg KT (1995). Pathogenesis of ascites tumor growth: vascular permeability factor, vascular hyperpermeability, and ascites fluid accumulation. Cancer Res.

[31] Byrne AT, Ross L, Holash J (2003). Vascular endothelial growth factor-trap decreases tumor burden, inhibits ascites, and causes dramatic vascular remodeling in an ovarian cancer model. Clin Cancer Res.

[32] Masquelier M, Vitols S (2004). Drastic effect of cell density on the cytotoxicity of daunorubicin and cytosine arabinoside. Biochem Pharmacol.

[33] Boissel N, Cayuela JM, Preudhomme C (2002). Prognostic significance of FLT3 internal tandem repeat in patients with *de novo* acute myeloid leukemia treated with reinforced courses of chemotherapy. Leukemia.

[34] Skovsgaard T (1978). Carrier-mediated transport of daunorubicin, adriamycin, and rubidazone in Ehrlich ascites tumour cells. Biochem Pharmacol.

[35] Boer H, Proost JH, Nuver J (2015). Long-term exposure to circulating platinum is associated with late effects of treatment in testicular cancer survivors. Ann Oncol.

[36] Aller SG, Yu J, Ward A (2009). et al. Structure of P-glycoprotein reveals a molecular basis for polyspecific drug binding. Science.

[37] Colabufo NA, Berardi F, Perrone MG (2010). et al. Substrates, inhibitors and activators of P-glycoprotein: candidates for radiolabeling and imaging perspectives. Curr. Top. Med. Chem.

[38] Wesolowska O (2011). Interaction of phenothiazines, stilbenes and flavonoids with multidrug resistance-associated transporters, P-glycoprotein and MRP1. Acta Biochim Pol.

[39] Xia WH, Zhao T, Lv J (2009). et al. Celecoxib enhanced the sensitivity of cancer cells to anticancer drugs by inhibition of the expression of P-glycoprotein through a COX-2- independent manner. J Cell Biochem.

[40] Jin W, Liu Y, Xu S (2010). UHRF1 inhibits MDR1 gene transcription and sensitizes breast cancer cells to anticancer drugs. Breast Cancer Res. Tr.

[41] Vrignaud P, Londosgagliardi D, Robert J (1986). Cellular pharmacology of doxorubicin in sensitive and resistant rat glioblastoma cells in culture. Oncology.

[42] El-Kareh AW, Secomb TW (2005). Two-mechanism peak concentration model for cellular pharmacodynamics of Doxorubicin. Neoplasia.

[43] Raijmakers R, Speth P, de Witte T (1987). Infusion-rate independent cellular adriamycin concentrations and cytotoxicity to human bone marrow clonogenic cells (CFU-GM). Br J Cancer.

[44] Skipper HE, Schabel FM, Wilcox WS (1964). Experimental evaluation of potential anticancer agents. XIII. On the criteria and kinetics associated with “curability” of experimental leukemia. Cancer Chemother Rept.

[45] Lalami Y, Klastersky J (2017). Impact of chemotherapy-induced neutropenia (CIN) and febrile neutropenia (FN) on cancer treatment outcomes: An overview about well-established and recently emerging clinical data. Crit Rev Oncol Hematol.

[46] Eskander RN, Tewari KS (2012). Impact of chemotherapy-induced neutropenia on survival in patients with breast, ovarian and cervical cancer: a systematic review. J Hematol Malig.

